# Hermetic Bags: A Short-Term Solution to Preserve High-Moisture Maize during Grain Drying

**DOI:** 10.3390/foods13050760

**Published:** 2024-02-29

**Authors:** Nileshwari Raju Yewle, Richard L. Stroshine, R. P. Kingsly Ambrose, Dieudonne Baributsa

**Affiliations:** 1 Department of Botany & Plant Pathology, Purdue University, West Lafayette, IN 47907, USA; nyewle@purdue.edu; 2 Department of Entomology, Purdue University, West Lafayette, IN 47907, USA; 3 Department of Agricultural and Biological Engineering, Purdue University, West Lafayette, IN 47907, USA; strosh@purdue.edu (R.L.S.); rambrose@purdue.edu (R.P.K.A.)

**Keywords:** postharvest storage, grain drying, PICS bags, germination, smallholder farmers

## Abstract

Maintaining maize quality while drying during a rainy season is a major challenge for smallholder farmers in developing countries. We conducted a study to evaluate the impact of temporarily storing wet maize of 18, 21, and 24% moisture content (m.c.) in hermetic Purdue Improved Crop Storage (PICS) and polypropylene (PP) woven (control) bags for 21 days. Oxygen and carbon dioxide concentrations were monitored, and m.c., germination, and visual mold were assessed. In PICS bags, oxygen dropped below 1% within 7, 11.5, and 21 days for maize at 24, 21, and 18% m.c., respectively. After 21 days, the m.c. of maize stored in PICS bags remained constant, but decreased in PP bags. Germination of maize in PICS bags decreased by 0.5, 6.2, and 95.5 percentage points for 18, 21, and 24% m.c., respectively. In PP bags, germination decreased by 17.5, 15.2, and 39.5 percentage points for the respective moisture levels. After 21 days of storage, visible mold was present on maize stored in PP bags at both 21 and 24% m.c. No mold was observed on maize stored in PICS bags, but a fermentation smell was released from maize at 21 and 24% m.c. The results indicate that maize can be effectively stored in PICS bags at 21% m.c. or below for 21 days with minimal germination loss or mold growth. These findings highlight the potential of using hermetic bags for short-term grain quality preservation just before and during drying. This new utility adds to the current use of hermetic bags for protection against pests during long-term storage. Hermetic bags’ dual functionality could significantly improve postharvest management on smallholder farms, thereby enhancing food and nutritional security and safety. Field testing is required in order to integrate this approach under smallholder farmers’ conditions (e.g., temperature, m.c., drying practices, etc.).

## 1. Introduction

Maize (*Zea mays*) is an important cereal crop for agri-food systems [1]. As a staple crop that can provide food security, maize is important in tropical and subtropical regions. This crop is grown several times a year based on climate, rainfall, temperature, soil type, availability of irrigation, and market demand. Depending on the region and growing season (major or minor), maize is harvested during or right before the rainy season [2]. Drying maize (field or home) during the rainy season is a major challenge encountered by small-scale producers in developing nations due to the limited availability of sunlight [3,4,5]. Managing grain dryness to ensure a sufficiently low m.c. is crucial for preserving maize quality [6]. Improper drying and storage of grain can reduce its usefulness for food and feed [7,8]. Therefore, drying is essential to maintaining grain quality during storage.

Smallholder farmers in developing countries employ various strategies to effectively manage wet maize for quality preservation. Common practices include leaving kernels on cobs during storage in granaries, piles/stacks, or hanging under roofs [9,10,11]. These methods promote air circulation, facilitating the continuation of the drying process [12]. However, these practices have been declining in recent years, mainly due to losses caused by pests and the increased risk of theft, as most of these methods store the maize outside of the household [13,14,15]. Given the challenges noted above and improved access to postharvest technologies (e.g., shellers), most smallholder farmers shell and store maize in polypropylene (PP) or gunny bags inside their homes. The choice of these bags is driven by their porous nature, allowing for continuous airflow and drying.

After harvest, small-scale producers heavily rely on the sunlight to dry their crops [4,13]. Sun-drying, although a simple and cost-effective method, has drawbacks such as labor requirements and lack of protection from animals [6,16]. Sun-drying is a relatively slow process that often stretches over several days, or even longer if there are consecutive days of rain or heavy cloud cover. On a daily basis, grain is moved in and out of the house to minimize theft and the impact of bad weather. This labor-intensive task involves grain redistribution at the beginning of the day, followed by spreading, frequent stirring, and collection in the evening [3,6]. A slow drying process can easily lead to quality deterioration [17]. Microbial activity can compromise maize quality if the grain is kept in the house for several days while waiting for optimal drying conditions. Loss of quality usually involves a notable decline in seed germination and/or the presence of microorganisms (such as mold and fungus), which can make the product unsuitable for consumption by humans and/or animals [18,19].

Maintaining grain quality during drying is paramount, as it directly affects food security, income, and safety. Germination is critical because most smallholder farmers in developing countries rely on harvested crops for seed for the next planting season. Temporary measures to safeguard maize until favorable weather conditions permit the resumption of the drying process emerge as a potential solution, not only to maintain grain quality, but also to reduce the labor involved. Studies have tested hermetic storage systems to maintain the quality of high-moisture maize during storage [20,21,22]. The results have shown that hermetic storage bags, including the Purdue Improved Crop Storage (PICS), prevent insect damage and slow fungal development. However, storing wet grain in hermetic bags can affect their quality, including loss of germination as well as qualities such as color change and fermentation [6,23,24,25,26].

Millions of smallholder farmers use hermetic bags to store grains and seeds of various crops, including maize, cowpea, common beans, rice, and sorghum [27]. Farmers store grains in hermetic bags for several months, sometimes extending storage until just before the next crop harvest or even beyond. By the subsequent harvest, these hermetic bags are typically emptied, primarily due to the use of stored grain for home consumption or sale. These empty hermetic bags present an opportunity because they can be used for other purposes. Investigating the feasibility of utilizing empty hermetic bags for the short-term storage of wet maize to preserve grain quality during drying is opportune. The role of temporary grain storage techniques and structures in preventing the quality loss of wet and dry grain has been explored in large facilities [28,29]. However, no studies have examined these options for smallholder farmers.

Research has demonstrated that temporarily holding high-moisture maize in hermetic jars has minimal effects on maize quality [30]. This study shows that wet maize at 18 and 21% m.c. can be hermetically stored for 14 and 7 days, respectively, without unacceptable loss of germination or mold development. Exploring whether hermetic bags used by smallholder farmers to store dried grain can be used to maintain the quality of wet maize is critical. This would allow for drying when optimal weather conditions are available, thereby expediting the overall drying process. The benefits would be twofold: (i) it would help to maintain maize quality during drying, and (ii) it would increase the utility of hermetic bags, transforming them into a multipurpose postharvest technology. Expanding the utility of hermetic bags in this way would make the innovation more cost-effective and attractive to smallholder farmers.

## 2. Materials and Methods

### 2.1. Maize Preparation

Maize (*Zea mays*, variety Becks 5994V2P) used in this study was provided by the Agronomy Center for Research and Education (ACRE) at Purdue University, West Lafayette, IN, USA, in October 2021. Maize cobs were shelled using a single-ear sheller that removed the kernels from the cobs using rubber rollers. The shelled maize was kept in plastic bags and stored at 4 °C for 5 weeks. The moisture content (m.c.) was 20.0% (wet basis) at harvest, but decreased to 19.6% upon removal from storage. Maize was then divided into three batches, with each batch either being rewetted using a drum roller to achieve a target m.c. of 21 or 24% or dried to 18% [30].

### 2.2. Experimental Setup

This study was carried out at the Postharvest Innovation for Crop Storage Laboratory, Department of Entomology at Purdue University, West Lafayette, IN, USA, from 19 November 2021 to 10 December 2021 (21 days). Samples of the maize were stored in small-sized hermetic PICS and PP bags (L of 18.7 cm by W of 10.5 cm) purchased from PPTL—Tanga in Tanzania. PICS bags capable of holding 25 kg were cut into these small-sized hermetic bags and sealed using an electrical heat sealer (Uline H-86 Impulse Foot Sealer; Pleasant Prairie, WI, USA). A PICS bag is a three-layer storage container made of two plastic liners, one within the other, fitted inside a woven PP bag [31]. Each small PICS (hermetic) and PP bag (non-hermetic) was filled with about 1.5 kg of maize. The open end of each layer of the two PICS inner liners and the PP bag was tied separately with a plastic zip tie. There were 4 replicates for each of the three m.c.s and storage conditions (hermetic and non-hermetic bags), for a total of 24 bags. All the bags were kept in a CARON Growth Chamber (model 6025-1; Caron Products & Services, Inc., Marietta, OH, USA) set at 25 ± 1 °C and 80% relative humidity (RH). Four bags from each m.c. were opened after 21 days.

### 2.3. Monitoring of Gas Composition

To enable gas measurement, each hermetic bag was fitted with a silicon rubber septum that protruded from the outer side of the bag. Both oxygen (O_2_) and carbon dioxide (CO_2_) were monitored at intervals of 12 h for 21 days using a Mocon^®^ portable O_2_/CO_2_ analyzer (Pac Check^®^ 325, Mocon Inc., Brooklyn Park, MN, USA). The Mocon needle was inserted into the interior of the hermetic bags through the septum. After each measurement, the septum was covered with adhesive tape to minimize gas leakage. For non-hermetic storage, the needle was inserted through the weaves of the PP bag near the opening.

### 2.4. Moisture Content, Temperature, and Relative Humidity

The m.c. of maize was measured using the oven drying method at a temperature of 103 ± 1 °C [32]. This involved assessing the weight before and after drying, with three 20 g grain samples subjected to a 72 h drying period. Temperature and relative humidity (RH) were collected every hour for 21 days using data loggers (Centor Thai, Rhino Research Group, Phichit, Thailand) placed in each hermetic and non-hermetic bag.

### 2.5. Germination and Shoot and Root Length

The germination rate was determined using the protocol developed by the International Seed Testing Association [33]. Samples from each replicate bag were prepared by placing four sets of 25 seeds between layers of moistened paper towels in Petri dishes (100 seeds). Each day for a week, kernels were checked for germination, with germination defined as a shoot length of at least 2 mm. After germination, ten randomly selected seedlings from each Petri dish were measured with digital calipers to assess the shoot (SL) and root lengths (RL). The average root and shoot lengths were calculated by summing the values and then dividing the total by ten. Out of 400 seeds tested, only 1 germinated from those stored in hermetic bags at a moisture content of 24%. Hence, we used the one seed to assess SL and RL.

### 2.6. Mold Assessment

At the end of the sampling period, each bag was opened, and 250 g of kernels were extracted from the center of the bag and four cardinal points using a 4-slot probe. Twenty-five maize seeds were randomly selected from the 250 g sample extracted from each bag. The seeds were placed into a Petri dish and then examined with the naked eye for signs of fungal growth. The Petri dishes containing the seeds were then taken to another lab in Purdue’s Entomology Department to further evaluate the seed coat, tip cap, and pedicel using a Leica S6 D Greenough stereo microscope with 10× magnification.

### 2.7. Data Analysis

The data were analyzed using the statistical package R (version 4.2.2). The linear mixed model was used to analyze the effects of m.c. on the oxygen depletion rate. A trend analysis was performed to develop a model for oxygen and carbon dioxide consumption across various m.c.s. A two-way analysis of variance (ANOVA) was conducted to examine differences among the three m.c.s (18, 21, and 24%) at the beginning and end of the experiment. Mean comparisons were made using the Student–Newman–Keuls (SNK) test. Microsoft Excel 2016 (Microsoft, Redmond, WA, USA) was utilized to generate all the graphs.

## 3. Results

### 3.1. Gas Composition

**Figure 1:**
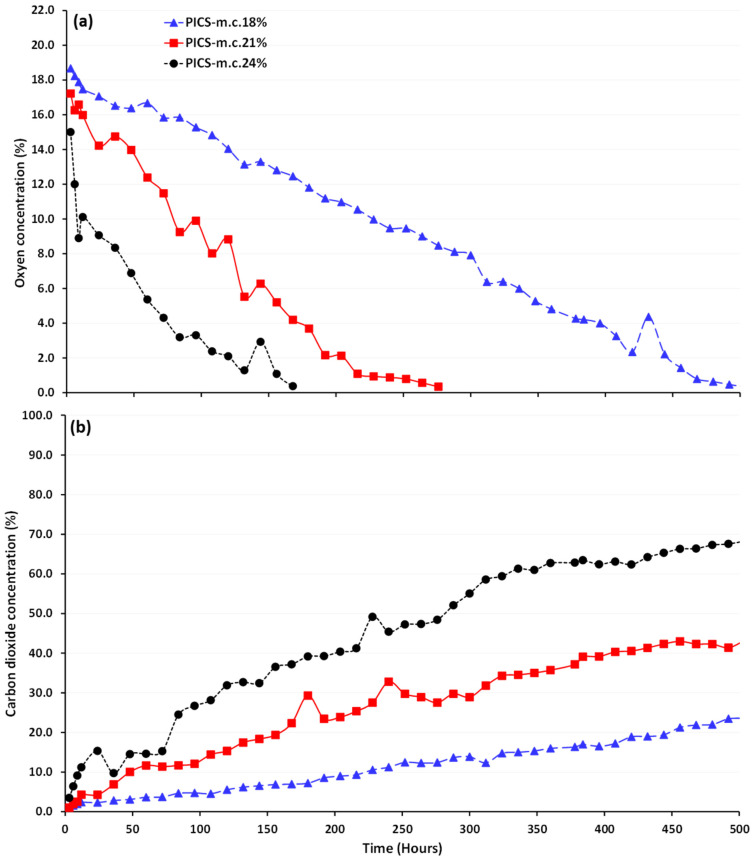
Levels of oxygen (**a**) and carbon dioxide (**b**) inside hermetic bags storing maize at the three m.c.s (18, 21, and 24%) when kept for 21 days in a growth chamber maintained at 25 °C and 80% relative humidity.

During the 21-day storage period, O_2_ levels in the PICS bags declined from ambient levels (21%) to 0%, while CO_2_ levels, initially non-detectable (0%), increased to as high as 62% in the bag containing maize at 24% m.c. (Figure 1). Anoxic conditions were reached within 168, 276, and 504 h inside PICS bags containing 24, 21, and 18% m.c., respectively, and remained there until the end of storage. Conversely, in the non-hermetic bags, O_2_ and CO_2_ levels remained at ambient conditions throughout the 21 days of maize storage. Tukey’s test revealed statistically significant differences in the levels of O_2_ (F = 1800; *p* = 0.001) and CO_2_ (F = 744.36; *p* = 0.001) among m.c. treatments in the hermetic bags. In addition, significant differences were observed in O_2_ and CO_2_ levels over time, with F = 3739.89 (*p* = 0.001) and F = 3365.89 (*p* = 0.001), respectively. Furthermore, a two-way ANOVA revealed statistically significant differences in the levels of O_2_ (F = 792.60; *p* = 0.001) and CO_2_ (F = 4642.92; *p* = 0.001) for the combination of storage time and m.c.

Linear model estimates for the fixed effects of m.c.s on O_2_ and CO_2_ levels are shown in Table 1. Significant statistical differences existed for all fixed effects, with *p* < 0.001.

The fixed effects pertaining to m.c. exhibited distinct patterns: negative for O_2_ consumption and positive for CO_2_ increase. Specifically, an m.c. increase from 18% to 21 or 24% corresponded to a notable decrease in O_2_ concentration of −5.50 to −9.91 percentage points, respectively. On the other hand, the CO_2_ concentration exhibited the opposite trend, with significant increases of 14.57 and 31.86 percentage points when m.c. rose from 18% to 21 or 24%, respectively.

The effects of m.c. on O_2_ depletion and CO_2_ increase inside hermetic bags were generalized using the equations shown in Table 2. Based on these equations, the O_2_ and CO_2_ concentrations in PICS bags at 18% m.c. followed linear trends. However, at 21 and 24% m.c., O_2_ and CO_2_ concentrations in PICS bags exhibited non-linear patterns that fit a second-order polynomial. The R^2^ values for the equations indicate that these models effectively describe the relationship between either O_2_ or CO_2_ concentration and time for the three m.c.s.

### 3.2. Moisture Content, Temperature, and Relative Humidity (RH)

A two-way ANOVA showed a statistically significant interaction between storage time and m.c. under hermetic and non-hermetic storage conditions (F = 111.32; *p* < 0.001). Subsequent analysis indicated a substantial change in m.c. within moisture treatments (F = 352.84; *p* < 0.001) and storage time (F = 313.07; *p* < 0.001). The initial m.c.s in both hermetic and non-hermetic bags closely aligned with the targeted levels of 18, 21, and 24%. However, after 21 days, the decreases in m.c. in the non-hermetic bags at 21 and 24% were statistically significant (Table 3). There were minimal differences in RH and temperature inside PICS and PP bags storing maize at different m.c.s (Figure S1). The RH recorded in the growth chamber during the entire storage period was maintained between 80 and 82%.

### 3.3. Seed Germination Assessment

The initial seed germination rates were consistent across treatments, but the rates changed after 21 days of storage (Table 3).

The germination exhibited variations within m.c. (F = 65.67; *p* < 0.001) and storage time (F = 233.11; *p* < 0.001). For the percentage of germination, the interaction between storage duration and m.c. was significant for both hermetic and non-hermetic storage conditions (F = 56.83; *p* < 0.001). Notably, for both hermetic and non-hermetic treatments, the most substantial decline in percent germination after 21 days of storage was observed in maize with an m.c. of 24%. In PICS bags, the germination declined by 0.5, 6.2, and 95.5 percentage points for m.c.s of 18, 21, and 24%, respectively. Conversely, the germination rates for maize stored in PP bags dropped by 17.5, 15.2, and 39.5 percentage points for the same respective m.c.s.

### 3.4. Seedling Growth (Root and Shoot Length)

Shoot and root lengths exhibited variability among treatments (Table 3) at 0 and 21 days. After 21 days of storage in PICS bags, there were no increases in shoot or root length compared to 0 days, except for the root length of maize at 21% m.c. Root length varied with both m.c. (within columns; F = 4.90; *p* = 0.0016) and storage time (within rows, F = 14.18; *p* = 0.0006). There was a statistically significant interaction between m.c. treatments and storage time for the root length (F = 7.27; *p* < 0.001). The shoot length varied within moisture treatments (columns, F = 7.93; *p* < 0.001) and storage times (rows, F = 9.15; *p* = 0.0046). For the shoot length, there was also an interaction between m.c. and storage time (F = 3.2; *p*= 0.0173).

### 3.5. Visual Assessment of Mold Growth

**Figure 2:**
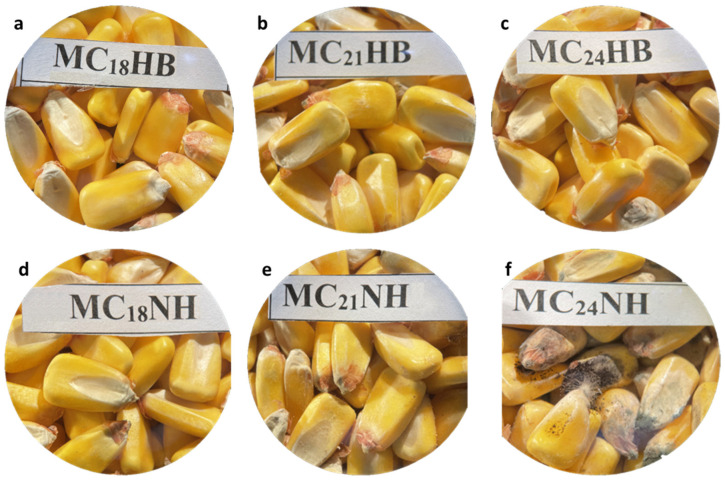
Stereomicroscope (10×)-magnified images for visual mold assessment of maize kernels kept in hermetic (**a**–**c**) and non-hermetic (**d**–**f**) bags at 18, 21, and 24% m.c., respectively, and stored for 21 days in a growth chamber maintained at 25 °C and 80% relative humidity.

**Figure 3:**
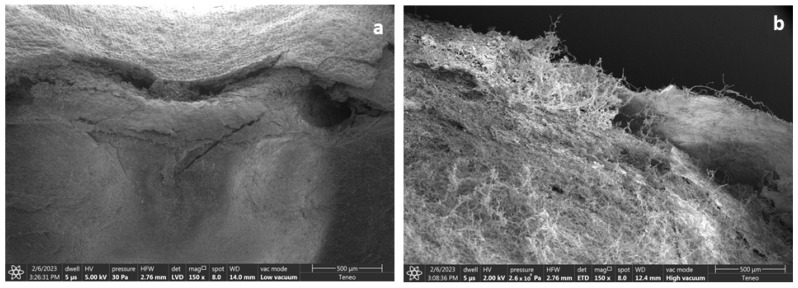
Photo of the kernel surface of maize kept at 24% m.c. (**a**) before and (**b**) after (21 days) storage in hermetic bags in a growth chamber maintained at 25 °C and 80% relative humidity. Image magnification (150×) by FEI Nova-Nano field emission SEM machine, with a low vacuum detector (LVD). Images taken at the Purdue Life Science Microscopy Facility (LSMF).

Mold growth significantly and negatively impacts the quality of grains and seeds when it occurs during storage. After 21 days, the maize kept at 21 and 24% m.c. in non-hermetic bags exhibited the most visible mold (Figure 2). Moreover, grain discoloration was evident for maize at 21 and 24% m.c. Non-hermetic bags presented additional challenges, including a powdery dust and an off odor, indicating maize spoilage. Conversely, maize stored at 18% in hermetic bags showed no apparent mold growth. However, mold was only visible under a microscope when maize was kept at 21 and 24% m.c. in hermetic bags (Figure 2 and Figure 3). This was accompanied by a smell of fermentation.

## 4. Discussion

This study aimed to assess the impact of hermetic storage of wet maize in PICS bags on the quality of the maize before and during drying. Following a 21-day storage period, maize kept in PICS bags maintained its original m.c. (18, 21, or 24%); however, it decreased in maize stored in PP bags at 21 and 24% m.c. Previous research has documented no changes in the m.c. of grain stored in hermetic bags after several weeks [20,30]. The change in m.c. observed in PP bags is not surprising, given that the grain moisture level in these containers is influenced by environmental conditions such as ambient temperature and RH [23,34]. This phenomenon is attributed to airflow through PP bags during storage [12].

### 4.1. Impact of Short-Term Hermetic Storage of Moist Maize on Germination and Seedling Growth

Hermetic bags effectively preserved maize germination when maize was stored at 18 and 21% for 21 days. The reduction in the germination of maize seeds stored in PICS bags was proportional to oxygen depletion rates, which were influenced by the m.c. of the stored maize. The more quickly the oxygen concentration decreased, the greater the loss of maize germination after 21 days of storage. In this study, the initial m.c. of the maize seed impacted the oxygen consumption rate in PICS bags. The rates of oxygen depletion and CO_2_ increase were faster for the higher m.c.s of 21 and 24%. There was about a doubling of the rates of oxygen depletion and CO_2_ increase as the m.c. increased by 3 percentage points from 21 to 24%. Anoxia, attained within a week of storage at 24% m.c., resulted in a total loss of germination. However, anoxia of the maize at 21% m.c. occurred within 13 days, but had minimal impact on seed viability after 21 days. Previous research has demonstrated that, during hermetic storage, the germination decreases more rapidly as the m.c. increases [21,30].

These patterns of loss in germination when wet maize is stored in hermetic bags can be attributed to the metabolic activity of maize seeds, consuming O_2_ through respiration and releasing CO_2_, leading to fermentation at higher m.c.s [35,36,37]. Fermentation of wet maize can occur unintentionally due to poor grain handling during drying or improper storage conditions. Hence, it is advisable to allow maize to dry to 21% or below before short-term storage in hermetic containers. This will preserve seed viability, as most small-scale producers depend on saved seeds for planting in subsequent growing seasons.

Research on the hermetic storage of maize at 21% m.c. revealed that germination, with minimal reduction, was maintained for only 7 days in jars [30]. Oxygen consumption and CO_2_ production were plotted over time for maize kept in jars [30] and PICS bags at three m.c.s (18, 21, and 24%) (current study). The oxygen consumption curves for maize stored in PICS bags and jars were almost identical at 18 and 24% m.c. However, O_2_ consumption was slower in PICS bags than in jars for maize at 21% m.c. The release of CO_2_ was similar for 18% m.c., but 5–20% lower in PICS bags for 21 and 24% m.c. Apparently, some additional respiratory activity occurred in the 21% m.c. jars that consumed oxygen. This unexpected respiratory activity of maize kept in jars at 21% m.c. indicates the possibility of mold growth, which can contribute to oxygen depletion [38].

The decline in maize germination within non-hermetic PP bags was more pronounced in maize stored at 24% m.c. The differences in germination among samples of maize stored at different m.c.s in PP bags could be attributed to slower mold growth at the lower m.c.s. Distinctions in mold growth among the PP bag samples are depicted in Figure 2d–f. Previous research has demonstrated that storage fungi have the potential to infect and damage embryos, thereby reducing seed germination [39]. Simultaneously, a decrease in m.c. was observed in grains stored in PP woven bags, aligning with findings from other studies [23]. This reduction in the m.c. of grain held at 21 and 24% may also explain the higher level of seed viability. These findings underscore the differential impact of storage conditions on percent germination, emphasizing the importance of both m.c. and the choice of storage bag for preserving seed viability over time.

While root and shoot length measurements were employed as indicators of seed vigor, no clear patterns were observed. In contrast to our findings, other studies have shown a negative impact of factors such as storage m.c. on seed vigor [40,41].

### 4.2. Impact of Short-Term Hermetic Storage of Moist Maize on Fungal Growth

Hermetic bags showed minimal fungal growth regardless of m.c. This absence of mold growth in PICS bags has been documented in previous studies where maize was stored for up to 75 days [21,22,30]. Because PICS bags are designed with multiple layers, they provide an adequate oxygen barrier and reduce the chances of mold and fungal growth. The substantial mold growth on maize stored in PP bags, particularly at 21 and 24% m.c., indicates the need to reduce the m.c. of wet maize before short-term storage in non-hermetic containers. *Aspergillus flavus*, a fungus that grows on maize and other crops at high m.c., can produce aflatoxin, a carcinogenic mycotoxin [42]. It can grow on maize before and after harvest, but can be prevented through proper drying and storage [17]. High-moisture maize (up to 21%) can be safely stored for one or two months under sealed conditions with self-regulated atmospheres that protect against microflora damage if fermentation and some loss of germination are allowable [21,22].

In this study, the high m.c. maize stored in PICS bags produced odors associated with fermentation. Various factors influence the fermentation of wet maize in hermetic bags, but it is predominantly microbial activity. Elevated moisture levels provide an optimal environment for the proliferation of bacteria and yeast [43], which initiate the fermentation process [24,44]. The consumption of oxygen under airtight storage conditions results in anaerobic conditions needed for fermentation, while the glucose and fructose found in the maize serve as substrates for the fermentation process. Microbial metabolism converts these sugars into ethanol and carbon dioxide. Although hermetic and non-hermetic storage methods have drawbacks, hermetic storage offers a better alternative because fermented maize can still be used for other purposes, such as animal feed, unlike grain infected with fungi. Achieving an m.c. below 21% before storing maize in hermetic bags is essential to prevent fermentation and mitigate its impact on maize quality.

### 4.3. Implication of Short-Term Storage of Wet Maize in Hermetic (PICS) Bags

After maize harvest, sun drying on a smallholder farm (before storage or sale) is a continuous and intermittent process that often requires several days, depending on the prevailing weather conditions. During drying in the sun, grain loses moisture over time, and is often kept in containers (e.g., woven bags) overnight or until weather conditions are favorable (e.g., sunny). These drying practices offer an opportunity to use hermetic bags for temporarily holding maize while drying instead of using PP woven bags. Hermetic bags would provide several benefits, including reduced labor and time needed for drying and handling grain under bad weather conditions. Grain temporarily stored in hermetic bags could be kept in houses or storage facilities for several days until optimal weather conditions are available to continue drying. These same hermetic bags could be used for subsequent storage of the dried maize, effectively preserving the dried grains from attack by insect pests and mold [30].

Though hermetic storage has the potential for short-term storage of maize (and other grains) during drying on smallholder farms, it is necessary to assess its performance in situ. Future research should explore the integration of short-term storage of wet maize into postharvest drying practices on smallholder farms. This would require assessing the optimal length of time for which wet grain can be temporarily stored in hermetic bags under various conditions (e.g., temperature and m.c.). Temperature and moisture content influence grain quality during storage [45,46,47,48]. Capacity building is necessary for integrating hermetic bags for short-term storage of moist maize into grain handling on smallholder farms. Farmers must learn proper grain handling practices in order to maximize the benefits of using hermetic bags for both short- and long-term storage. By using hermetic bags for short-term storage, smallholder farmers would benefit by improving the quality of grain they store and consume. Further, this would also allow smallholder farmers to take advantage of market opportunities by supplying clean and safe grain to consumers and processors.

## 5. Conclusions

This research demonstrated that hermetic bags can maintain the quality of wet maize stored for 21 days. The germination rate stayed consistently above 90% for maize stored at either 18 or 21% m.c. for 21 days. The initial m.c. of the maize has a significant impact on oxygen consumption and the germination rate of the seeds. Hermetic PICS bag storage of maize seeds at 24% m.c. caused oxygen to deplete within a few days, resulting in a complete loss of germination. Although there was minimal fungal growth in the wet maize stored in PICS bags at 21 and 24% m.c., the grain removed from the bags after 21 days had a distinct odor of fermentation. These results indicate the possibility of using hermetic bags to maintain grain quality during drying by protecting it from pests and fungi. Utilizing hermetic bags for a combination of short- and long-term storage would improve postharvest grain management and food and nutrition security on smallholder farms. The integration of hermetic bags as a temporary solution for storing wet maize on smallholder farms requires additional research in order to better understand (i) the role of temperature and m.c. in oxygen consumption and grain quality, such as germination and microbial activity; (ii) how this technique could be part of typical postharvest drying practices used on smallholder farms; and (iii) the economics of using hermetic bags as a dual storage solution. Utilizing hermetic bags as both a short- and long-term storage solution would contribute to the sustainable use of resources on smallholder farms.

## Figures and Tables

**Table 1:** Linear mixed model estimates of the fixed effects of moisture content (m.c.) on oxygen (O_2_) depletion and carbon dioxide (CO_2_) rise in hermetic bags storing maize at 18, 21, or 24% m.c. for 21 days. Hermetic bags were kept in a growth chamber maintained at 25 °C and 80% relative humidity.

Parameter *	Estimate	SEM ^a^	*t*	*p*-Value	LCI ^b^	UCI ^c^
Intercept	23.18	0.47	49.17	<0.001	22.25	24.10
O_2_ at PICS-m.c.18%	0	0				
O_2_ at PICS-m.c.21%	−5.50	0.21	−25.75	<0.001	−5.92	−5.08
O_2_ at PICS-m.c.24%	−9.91	0.24	−40.43	<0.001	−10.39	−9.42
Intercept	−13.59	1.80	−7.51	<0.001	−17.95	−9.24
CO_2_ at PICS-m.c.18%	0	0				
CO_2_ at PICS-m.c.21%	14.57	0.64	22.55	<0.001	13.31	15.84
CO_2_ at PICS-m.c.24%	31.86	0.64	49.31	<0.001	30.60	33.13

* Degree of freedom for parameters O_2_ = 368 and CO_2_ = 540. ^a^ SEM: standard error of means; *t* value; ^b^ LCI = lower confidence interval; ^c^ UCI = upper confidence interval.

**Table 2:** Relationship between oxygen and carbon dioxide concentration (y) at any time (x) in hermetic bags storing maize at the three moisture contents (18, 21, or 24%) when kept for 21 days in a growth chamber maintained at 25 °C and 80% relative humidity.

Treatment	Equation *	R^2^ Value
O_2_ at PICS-m.c.18%	y = −0.0366x + 18.4	0.995
O_2_ at PICS-m.c.21%	y = −0.0001x^2^ − 0.0994x + 17.484	0.988
O_2_ at PICS-m.c.24%	y = 0.0005x^2^ − 0.1451x + 12.597	0.935
CO_2_ at PICS-m.c.18%	y = 0.0429x + 0.7439	0.989
CO_2_ at PICS-m.c.21%	y = −0.0001x^2^ + 0.1312x + 2.0265	0.983
CO_2_ at PICS-m.c.24%	y = −0.0002x^2^ + 0.2208x + 5.3854	0.988

* y is the oxygen or carbon dioxide concentration level at time x.

**Table 3:** Germination (%), shoot and root lengths, and moisture contents of maize seeds stored in hermetic and non-hermetic bags after 21 days in a growth chamber maintained at 25 °C and 80% relative humidity.

Treatments	Germination %		Shoot Length (mm)		Root Length (mm)		Moisture Content (%)	
	0 Day	21 Days	0 Day	21 Days	0 Day	21 Days	0 Day	21 Days
PICS-m.c.18%	99.5 aA *	99.0 aA	9.44 bA	13.92 bA	55.57 aA	64.97 aA	17.59 cA	17.67 cA
PICS-m.c.21%	97.24 aA	91.0 abA	15.31 abA	21.85 aA	46.51 aB	70.73 aA	21.03 bA	20.87 bA
PICS-m.c.24%	95.5 aA	0.0 dB	14.61 abA	11.75 bA ^&^	46.18 aA	61.09 abA ^&^	24.08 aA	24.07 aA
PP-m.c.18%	99.5 aA	82.0 bB	10.33 abA	14.97 bA	55.68 aA	63.36 aA	17.59 cA	16.98 cdA
PP-m.c.21%	97.24 aA	82.0 bA	16.48 aA	18.04 abA	47.51 aA	46.87 bA	21.03 bA	16.67 dB
PP-m.c.24%	95.50 aA	56.0 cB	15.11 abA	15.16 abA	57.93 aA	45.58 bA	24.08 aA	17.42 cdB

* In the same column (lower case) or the same row (upper case) for a given variable, means with the same letters are not statistically significant at *p* = 0.05; ^&^ data collected from only 1 germinated seed out of 400 tested.

## Data Availability

The original contributions presented in the study are included in the article/supplementary material, further inquiries can be directed to the corresponding author.

## Supplementary Materials

The following supporting information can be downloaded at: https://www.mdpi.com/article/10.3390/foods13050760/s1, Figure S1. The mean relative humidity (RH) and temperature (Temp) of maize seeds kept in hermetic (a) and non-hermetic (b) bags at 18, 21, and 24% moisture for 21 days in a growth chamber maintained at 25 °C and 80% relative humidity. Data for 24% of non-hermetic bags are missing because the dataloggers malfunctioned at high relative humidity.
