# Supplementary material for: Hermetic Bags: A Short-Term Solution to Preserve High-Moisture Maize during Grain Drying
Source: Foods. 2024 Feb 29;13(5):760. doi: 10.3390/foods13050760 (PMC10930799; doi:10.3390/foods13050760)
Supplement: Supplementary file 1 [file foods-13-00760-s001.zip › foods-2820369-supplementary.pdf]

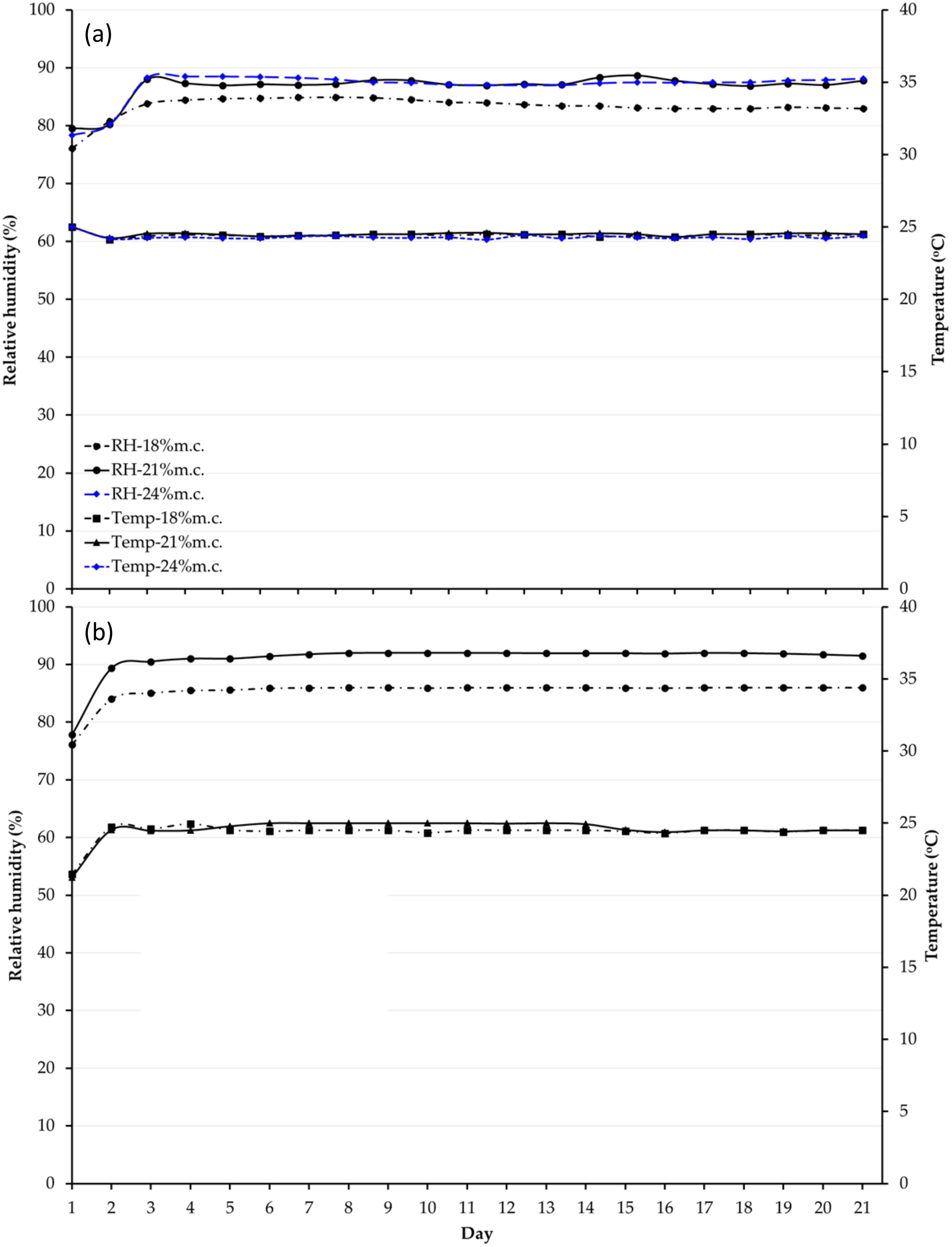

Figure S1. The mean relative humidity (RH) and temperature (Temp) of 18, 21, and 24% moisture of maize seeds stored in (a) hermetic and (b) non-hermetic bags for 21 days in a growth chamber maintained at 25°C and 80% relative humidity. Data for 24% of non-hermetic bags is missing because the dataloggers malfunctioned at high relative humidity.
